# Comparative Analysis of ANDE 6C Rapid DNA Analysis System and Traditional Methods

**DOI:** 10.3390/genes11050582

**Published:** 2020-05-22

**Authors:** Michele Ragazzo, Stefano Melchiorri, Laura Manzo, Valeria Errichiello, Giulio Puleri, Fabio Nicastro, Emiliano Giardina

**Affiliations:** 1 Department of Biomedicine and Prevention, Tor Vergata University of Rome, 00133 Rome, Italy; michele.ragazzo@uniroma2.it (M.R.); smelchiorri.sm@gmail.com (S.M.); laura.manzo@uniroma2.it (L.M.); valeria.errichiello@uniroma2.it (V.E.); giuliopuleri@gmail.com (G.P.); 2 Austech, 00153 Rome, Italy; f.nicastro@austech-italy.com; 3 Genomic Medicine Laboratory UILDM, IRCCS Santa Lucia Foundation, 00179 Rome, Italy

**Keywords:** forensic DNA typing, rapid DNA analysis, short tandem repeats, electropherogram, ANDE chip

## Abstract

Rapid DNA analysis is an ultrafast and fully automated DNA-typing system, which can produce interpretable genetic profiles from biological samples within 90 minutes. This “swab in—profile out” method comprises DNA extraction, amplification by PCR multiplex, separation and detection of DNA fragments by capillary electrophoresis. The aim of study was the validation of the Accelerated Nuclear DNA Equipment (ANDE) 6C system as a typing method for reference samples according to the ISO/IEC 17025 standard. Here, we report the evaluation of the validity and reproducibility of results by the comparison of the genetic profiles generated by the ANDE 6C System with those generated by standard technologies. A quantity of 104 buccal swabs were analyzed both through the ANDE 6C technology and the traditional method (DNA extraction and quantification, amplification and separation by capillary electrophoresis). Positive typing was observed in 97% of cases for ANDE 6C technology with only three buccal swabs failing to reveal interpretable signals. Concordance was determined by comparing the allele calls generated by ANDE 6C and conventional technology. Comparison of 2800 genotypes revealed a concordance rate of 99.96%. These results met the ISO/IEC 17025 requirements, enabling us to receive the accreditation for this method. Finally, rapid technology has certainly reached a level of reliability which has made its use in laboratories of forensic genetics a reality.

## 1. Introduction

Forensic DNA analysis plays an important role not only in criminal investigations but also assisting identification of victims in large-scale disasters. The accuracy of current methods of forensic DNA typing allows certain identification in the large majority of cases. The analysis of DNA forensic sample requests standardized and shared protocols through ultra-sensitive methods [1,2]. The need for shared methods is also managed and assessed through the accreditation of laboratories according to the UNI CEI EN ISO/IEC 17025 standard, which certifies their competence by ensuring the reliability of the results. It also promotes cooperation between laboratories and other bodies by generating wider acceptance of results between countries. To date, DNA profiles can be included in the national DNA database only if they have been obtained in certified laboratories in accordance with UNI CEI EN ISO/IEC 17025 standard [3]. According to these standards a number of minimal criteria need to be satisfied: separated working areas for every analytical phase (the tested material is moved following a unidirectional flow with the purpose of avoiding any possible cross-contaminations); the use of a number of validated instruments; and the presence of highly qualified technical analysts [4]. The time needed for the completion of such standard forensic DNA test may last several hours or even days and this period could be too long in the cases of illegal human trafficking, terrorist and criminals identification, and immigration and border situations [5]. 

The integration of all analytical steps into a single instrument can accelerate and simplify the completion of the workflow, reducing time and costs. Recently some fully automated rapid DNA systems have been developed [6]. Here, we provide the results of our recent accreditation of the ANDE 6C (Accelerated Nuclear DNA Equipment) Rapid DNA Identification System for the typing of reference samples [7,8,9]. 

The Rapid DNA Act of 2017 allows the implementation of rapid DNA in police station and the National DNA Index System (NDIS) approval is an essential requirement. Furthermore, the ANDE System generates DNA profiles that can be used to automatically search the FBI’s DNA database using the Rapid DNA Index System (RDIS) [10]. The ANDE 6C instrument is able to analyze 27 loci (FlexPlex27 Amplification kit, ANDE Corporation, Longmont, CO, USA) in accordance with expanded sets of core STR (Short Tandem Repeat) loci [11,12,13]. Use of the analytical microchip has numerous advantages over the conventional technology, including execution speed, portability of the instrument, decreasing of potential contamination and simplicity of the analysis execution. 

The compact size and portability of this instrument make this approach applicable not only for highly specialized forensic laboratories, but also when required at police stations, scenes of crime, cross-border points or wherever fast and reliable DNA identification is needed [14].

Here, we report results concerning the validity, robustness and reliability of genetic profiles generated by the ANDE 6C Rapid DNA Analysis System in comparison with those generated using validated and conventional DNA analysis procedures (semiautomated DNA extraction, PCR multiplex amplification and capillary electrophoresis using ABI 3130 DNA Analyzer, ThermoFisher Scientific, Waltham, MA, USA). These results led us to receive the accreditation of this system according to the ISO/IEC 17025:2017—category III, covering tests carried out by laboratory staff in places outside the site of the testing laboratory.

## 2. Materials and Methods 

This research study enrolled 104 volunteer donors who each contributed two buccal swabs, providing a total of 208 samples. These samples were utilized to perform a concordance study between the rapid and traditional method. Subsequently, our donors offered an additional 35 buccal swabs in order to perform repeatability and reproducibility studies. Written informed consent for the genetic analysis was obtained from all the subjects [15,16]. One sample of each donor was collected on ANDE swabs and it was processed using the ANDE 6C Rapid DNA System (ANDE Corporation, Longmont, CO, USA) utilizing the FlexPlex27 kit according to manufacturer’s recommendations [13]. The ANDE swab is essentially the Bode SecureSwab2 (ANDE Corporation, Longmont, CO, USA), a cotton swab with desiccant for storage. The main difference is that the ANDE swab cap contains an integrated RFID chip for sample tracking. The integrated ANDE Expert System was used for the allele calling and data interpretation [13]. The second sample of each donor was processed applying the standard laboratory protocol in order to conduct a comparative analysis between these analytical methods. DNA was extracted from the buccal swabs with the MagPurix kit (Zinexts Life Science Corporation, New Taipei City, Taiwan) according to the manufacturer’s instructions [17]. The Quantifiler^®^ Duo DNA Quantification kit (Thermo Fisher Scientific, Waltham, MA, USA) was used was used to evaluate the concentration and the quality of the extracted DNA and for the amplification, we used the GlobalFiler™ PCR Amplification Kit (Thermo Fisher Scientific, Waltham, MA, USA) following the manufacturer’s protocols. The separation and detection were conducted with an Applied Biosystems^®^ 3130xl Genetic Analyzer (Thermo Fisher Scientific, Waltham, MA, USA) according to manufacturer’s protocols [12,18]. The collection and the analysis of the data obtained by capillary electrophoresis were assessed by GeneMapper^®^ software v.1.4 (Applied Biosystems, Waltham, MA, USA) [19,20]. To evaluate concordance, conventional DNA testing and allele calls were compared to the results of the ANDE System [12]. 

### 2.1. Ethical Committee Aproval

These investigations were carried out following the rules of the Declaration of Helsinki of 1975 revised in 2013. According to point 23 of this declaration, an approval from the ethics committee of Santa Lucia Foundation ethical committee (protocol number: Prot. CE/PROG.650 01-03-18). This ethics committee has also approved the written informed consent.

### 2.2. ANDE 6C Rapid DNA Analysis System

The ANDE 6C Rapid DNA Analysis System consists of the ANDE Swab, the ANDE chip and the ANDE instrument. The ANDE chip is a fully integrated lab-on-a-chip that performs all steps required for STR analysis (DNA extraction, STR amplification, electrophoretic separation and detection of amplified PCR products) in less than 90 min [21]. It contains all reagents on board, either in a freeze-dried form or in liquid form. The ANDE chip is closed and it is single-use to minimize the possibility of contamination. There are two types of ANDE chip: A-chip, which processes up to five samples simultaneously and is designed for use with buccal swabs, and I-chip, which is designed for low template DNA (LT-DNA) samples and is able to process up to four samples simultaneously. The forensic workflow within the chip can be grouped into three modules: Module 1—DNA purification, Module 2—STRs amplification, and Module 3—electrophoretic separation and detection [22].

The FlexPlex27 system is a multiplex assay that enables the analysis of the expanded CODIS (Combined DNA Index System) core loci and all additional STR loci required for international databasing. The assay contains 23 autosomal loci (D1S1656, D2S1338, D2S441, D3S1358, D5S81, D6S1043, D7S820, D8S1179, D10S1248, D12S391, D13S317, D16S539, D18S51, D19S433, D21S11, D22S1045, FGA, CSF1PO, Penta E, TH01, vWA, TPOX, and SE33), amelogenin, and three Y-chromosomal loci (DYS391, DYS576, and DYS570) [13].

### 2.3. Reliability of ANDE 6C Rapid DNA Analysis System

The reliability of the ANDE 6C Rapid DNA Analysis System was evaluated through genotyping success rate, correlation of genotypes between two analytical methods (rapid and traditional), the signal intensity reported in relative fluorescence units (RFUs) for each locus, peak height ratio (PHR) at each locus, precision and resolution analysis (maximum deviation reported in terms of base pair), and precision in terms of repeatability and reproducibility. The signal strength for each locus was calculated for the heterozygous profiles by summing the signal strengths of all called alleles within the locus and dividing by two. The heterozygous peak height ratio per locus was calculated for each of the profiles by dividing the height of the smaller peak by the height of the larger peak.

The inter-run precision was calculated by determining the fragment sizes’ (expressed in base pair) standard deviation per each allele of the allelic ladder samples (one allelic ladder is run per biochips). 

The inter-run precision was calculated through a comparison of the fragment sizes (reported as base pair and two decimals) of each allele in twenty-one allelic ladder. Each allele was typed 21 times generating medium values and standard deviation.

The resolution was calculated from the 18 synthetic DNA fragments included in ILS for the 104 profiles with passing internal lane standard (ILS), using the following formula (Equation (1)):

(1) R=2(t2−t1)/(w1+w2)

where t: migration time, w: baseline width [23].

The ILS runs with each sample and contains 18 synthetic DNA fragments. 

### 2.4. Repeatability and Reproducibility

The precision in terms of repeatability was evaluated by analyzing five replicates of the same sample, from the same qualified operator, in a single analytical session, with the same biochip and the same ANDE 6C instrument. Therefore, genotype concordance was evaluated between the five replicas and with respect to the reference sample [24,25,26,27].

The precision in terms of reproducibility was evaluated performing the analysis of ten samples (ten buccal swabs) already analyzed by the traditional method, varying the place of execution (tests performed both inside and outside laboratory) and at different temperature and humidity conditions. These parameters were monitored through an environmental thermo-hygrometer calibrated by an accredited center, to guarantee the metrological traceability as foreseen by the laboratory quality system (QS) [24,25,26,27,28].

In particular, ten buccal swabs were processed in three different environmental conditions through the rapid approach: within the Forensic Genetics Laboratory of the Tor Vergata University; outside the laboratory testing areas with temperatures ranging from 29.2 °C to 31.5 °C and humidity ranging from 27.1% to 30.0%; outside the University with temperatures ranging from 14.3 °C to 27.2 °C and humidity ranging from 29.7% to 72.1%. To undertake this analysis, the ANDE 6C instrument, the samples and reagents were transported by car for 23 km. Furthermore, the precision was evaluated by the allelic correspondence between the ten genetic profiles obtained with the ANDE 6C Rapid DNA System in the three different test conditions and through comparison of the results with those previously typed with the traditional method [24,25,26,27].

## 3. Results and Discussion

### 3.1. Genotyping Success Rate and Concordance

**Figure 1:**
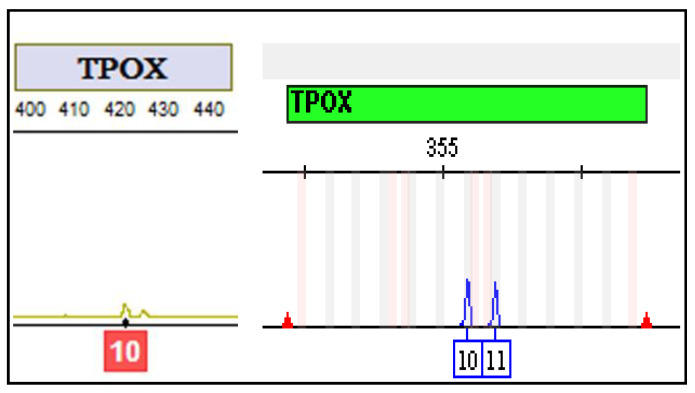
The allelic dropout at TPOX marker.

**Figure 2:**
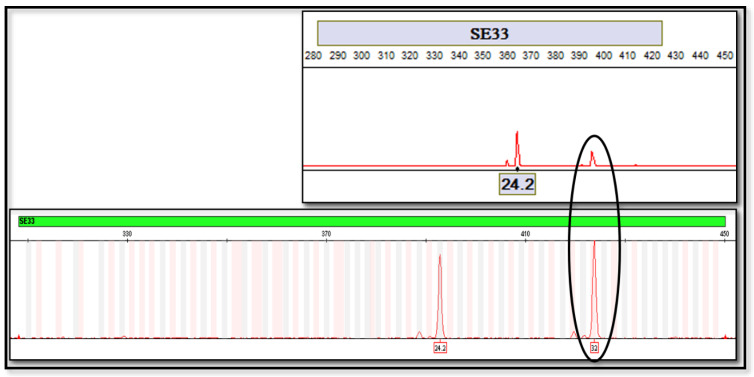
The off-ladder at the SE33 marker.

The ANDE 6C Rapid DNA Analysis System successfully analyzed 97% of biological samples. Only three buccal swabs failed to reveal interpretable signals. Concordance was determined by comparing the allele calls generated by ANDE 6C with the DNA profile of the same person produced by the conventional technology. The alleles called by the ANDE 6C Rapid DNA Analysis System had 99.96 % correlation. Of an estimated total of 2800 markers analyzed by the ANDE DNA Rapid System, only two of them were not correctly typed. A single allelic dropout was detected at the TPOX marker (Figure 1) in the profile generated with the ANDE 6C System, while it was properly called with the standard protocol. An off-ladder was highlighted at the SE33 marker (Figure 2).

These results indicated a variability of intermarkers and inter-fluorescence in terms of signal strength with respect to that seen in the current technology. The average peak height ranged from 2850 RFU for the D1S1656 marker to 11215 RFU for the TH01 marker. 

**Figure 3:**
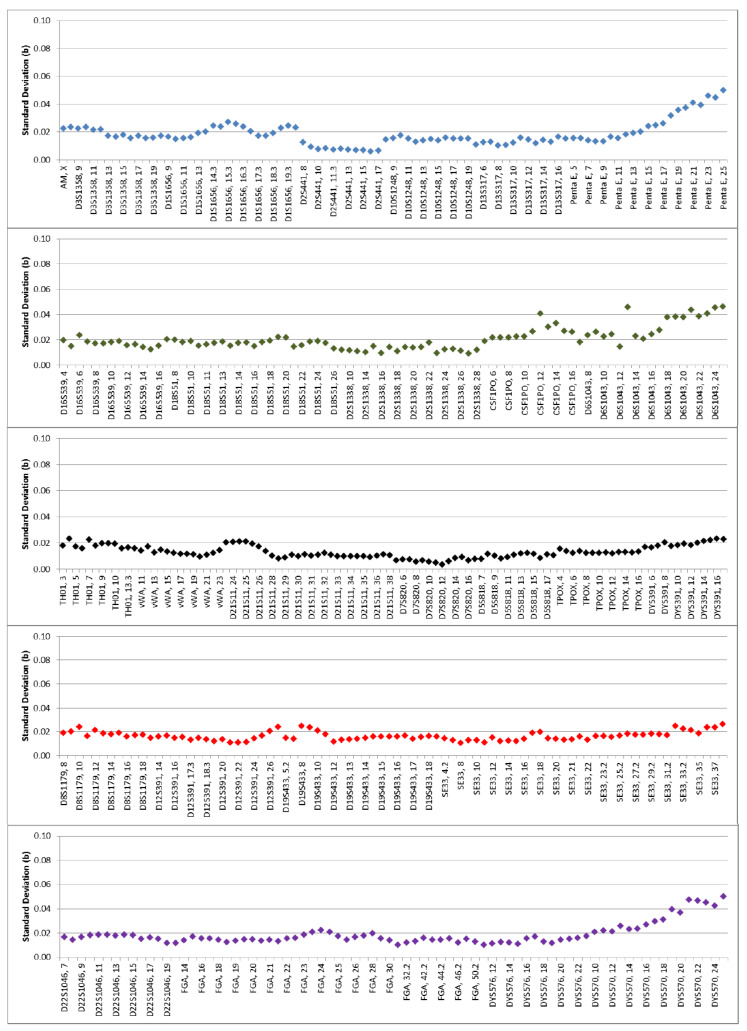
Inter run precision.

The average peak height ratio ranged from 0.712 for the SE33 marker to 0.849 for the D1S1656 marker. The mean molecular weight (bp) and relative standard deviation was compared between each allele in the 21 allelic ladders run. It ranged from 0.004 bases at the D7S820 marker to 0.050 bases at the DYS570 marker. The variation at the three standard deviations ranged from 0.012 bases to 0.140 bases and were well within the acceptable target value of 0.5 bases (Figure 3).

**Figure 4:**
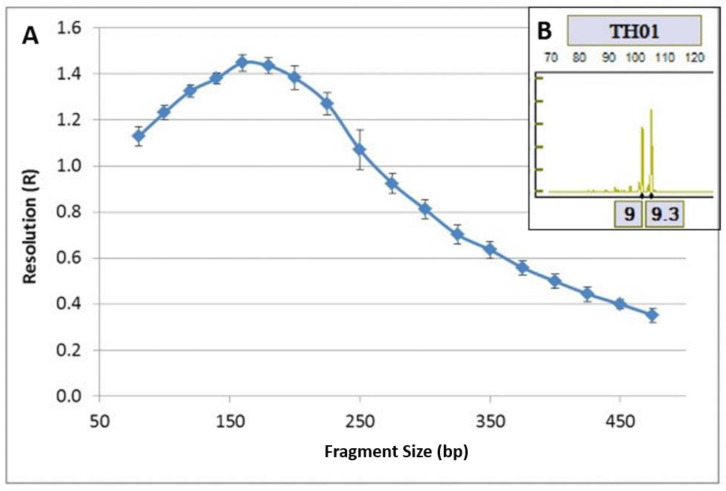
(**A**) Resolution calculated from internal lane standard (ILS) of 104 samples, (**B**) Microvariant detection example—3 bp microvariant at TH01.

Resolution value ≥ 0.2 indicates a single base pair resolution. The resolution based on these samples shows that the system is capable of correctly genotyping markers, independently of their size. The *R*-values remain inside the established resolution ranges and it is now considered standard in all laboratories (Figure 4A). The ANDE instrument demonstrated single base pair resolution as shown by correct differentiation of TH01 alleles, 9 and 9.3 (Figure 4B).

### 3.2. Precision in Terms of Repeatability and Reproducibility

In the repeatability study, the five replicates showed an allele correspondence of 100%, with the genetic profile obtained through the traditional method. We obtained 100% concordance for all replicates.

Reproducibility of ten samples in three different conditions was 100%. Full profiles with the expected genotypes were obtained for all samples. In particular, all the tested samples provided a genotyping success rate and concordance rate of 100%, regardless of environmental conditions (i.e., temperature, humidity, inside/outside the laboratory). The average peak height ratio ranged from 0.3617 for the Penta E marker to 0.9982 for the FGA marker.

## 4. Conclusions

The ANDE 6C Rapid DNA Analysis System successfully analyzed 97% of biological samples regardless of temperature and humidity conditions, use internally or externally, and before or after transportation. Only three buccal swabs failed to reveal interpretable signals.

The concordance study of 2800 markers revealed a value of 99.96%. No incorrect genotype calls were detected. A single allelic dropout was detected at the TPOX marker in the profile generated with the ANDE 6C System and a single off-ladder was highlighted at the SE33 marker.

All allelic calls showed optimal signal strength and balanced profile for heterozygous markers. 

Based on these results, the ANDE 6C Rapid DNA Analysis demonstrated its robustness and reliability to determine correct DNA profiles from reference samples (buccal swabs).

The quality of the results obtained from this project also made it possible to develop an internal test method and a quality management system necessary for the accreditation of mobile laboratories according to the EN ISO/IEC 17025: 2017 standard (category III) [29].

In conclusion, this project demonstrates that the ANDE 6C System is robust, reliable, and is suitable for use in human identification for forensic purposes from a single source of DNA. Presently it is used in our laboratory for testing reference samples. 

Rapid technology has certainly reached a level of reliability which has made its use in laboratories of forensic genetics a reality. The portability of this system has the potential to extend the use of genetic typing for forensic use. This technology presents several advantages in terms of cost and time. In particular, the cost of the rapid DNA method is less than that of the conventional laboratory—when the true cost of the laboratory methodology is included (laboratory and overhead). With respect to time, one instrument processes five samples in 96 min, for a throughput of 25 samples/day (assuming 5 runs/day). The reagent cost of a single rapid sample is approximately 2–3 times that for traditional methods. In particular a traditional profile costs about EUR 80 while a rapid DNA profile costs approximately EUR 200. 

The use of this system directly in public security offices, at borders and in mobile laboratories will allow the identification of subjects with extreme rapidity and will be able to guarantee genetic typing in complex locations.
